# Glycolytic Metabolic Remodeling by the Truncate of Glioma-Associated Oncogene Homolog 1 in Triple-Negative Breast Cancer Cells

**DOI:** 10.7150/jca.72793

**Published:** 2022-08-08

**Authors:** Su Hyun Lee, Ji Sun Lee, Jae Hyeon Park, Sungpil Yoon, Kwang Youl Lee, Hyung Sik Kim

**Affiliations:** 1 School of Pharmacy, Sungkyunkwan University, 2066, Seobu-ro, Jangan-gu, Suwon, 16419, Republic of Korea.; 2 College of Pharmacy, Chonnam National University, Yongbong-ro, Buk-gu, Gwangju, 61186, Republic of Korea.

**Keywords:** autophagy, glioma-associated oncogene, glycolytic rates, hedgehog (Hh) signaling, TNBC

## Abstract

Hedgehog (Hh) signaling pathway plays an essential role in embryonic development, tissue regeneration, and stem cell renewal. In particular, terminal effectors of the Hh signaling pathway are associated with the regulation of glioma-associated oncogene homolog 1 (GLI1) transcription factors. Overexpression of GLI1 is closely associated with poor prognosis in breast cancer. The Hh-GLI1 signaling pathway is activated and participates in the tumorigenesis and progression of breast cancer, especially in the aggressive subtype of triple-negative breast cancer (TNBC). However, the role of GLI1 in regulating TNBC metabolism remains unclear. This study aimed to explore the functional role of GLI1 in glycolytic metabolism in TNBC. Immunohistochemical analysis of GLI1 expression in a tissue microarray revealed significant correlations between GLI1 expression and advanced tumor stage and grade. GLI1 expression levels were drastically increased in MDA-MB-231 cells compared to those in other cell lines. Inhibition of GLI1 expression using GLI1 small interfering RNA (siRNA) in MDA-MB-231 cells resulted in a significant reduction in cell proliferation and induced cell cycle arrest at the G1 phase. Furthermore, GLI1 downregulation significantly reduced the expression of glycolysis-regulated proteins. GLI1 knockdown resulted in reduced glycolytic rates and extracellular lactate levels. Moreover, metabolic stress after GLI1 knockdown activated the energy sensor, adenosine monophosphate-activated protein kinase, which subsequently resulted in autophagy induction. In conclusion, this study indicates that targeting GLI1 reprograms the tumor glucose metabolism to suppress breast cancer cell growth and proliferation.

## Introduction

Breast cancer is the second most common and deadliest cancer in women worldwide. The most aggressive subtype is triple-negative breast cancer (TNBC), which accounts for approximately 15-20% of all cases. TNBC is characterized by a lack of the estrogen receptor (ER), progesterone receptor (PR), and human epidermal growth factor receptor 2 (HER2). It affects the younger patient population, metastasizes at a higher rate than other subtypes, and has a poor prognosis 1, 2. Owing to its heterogeneity, the development of targeted therapies for TNBC has largely been unsuccessful 3. Although chemotherapy significantly improves the clinical outcomes of patients with TNBC, recurrence rates remain relatively high, and TNBC tumors often develop resistance to chemotherapeutic agents 4, 5. Therefore, considering the limited treatment options and aggressive phenotypes of TNBC, it is crucial to improve our understanding of the features of TNBC and discover potential therapeutic targets to aid in its treatment.

The Hedgehog (Hh) signaling pathway is involved in many physiological and pathological processes, including cancer, adipocyte differentiation, diabetes, and obesity 6, 7. In particular, Hh signaling has been implicated in tissue patterning during embryonic development, tissue repair, and epithelial-to-mesenchymal transition 8, 9. The binding of Hh ligands (Sonic hedgehog (Shh), Indian hedgehog, and Desert hedgehog) releases the inhibitory effect of patched (Ptch), a transmembrane receptor, on smoothened (Smo), also located on the cell membrane. The signaling cascade initiated by Smo leads to the activation and nuclear localization of the transcription factors of the zinc finger family, glioma-associated oncogene (GLI) (GLI1, GLI2, and GLI3), which drive the expression of Hh target genes mostly involved in proliferation, survival, and angiogenesis 10, 11. Alterations in the Hh signaling pathway result in tumorigenesis and tumor growth in breast cancer 12. In particular, elevated Shh expression in TNBC correlates with inferior overall survival 13.

Among GLI proteins, GLI1 mainly acts as a transcriptional activator because of the absence of an N-terminal repressor domain 10. In addition to the canonical activation of GLI1 by the Hh-Ptch-Smo route, growing evidence indicates a Smo-independent stimulation of GLI1 activity in cancer, including the K-Ras, transforming growth factor-β 14, and phosphoinositide 3-kinase (PI3K)/protein kinase B (Akt)/mammalian target of rapamycin (mTOR) pathway 15. Non-canonical activation of GLI1 seems to account for the low therapeutic efficacy and resistance of clinically available Smo inhibitors (vismodegib and sonidegib) in cancer treatment 16. Thus, inhibiting GLI1-mediated transcription seems to be an alternative strategy for successful cancer therapy, making it necessary to explore the unknown role of GLI1 in cancer.

Aerobic glycolysis (Warburg effect) is a key metabolic phenotype in malignant tumors. Cancer cells tend to produce energy via glycolysis rather than oxidative phosphorylation (OXPHOS), regardless of oxygen availability 17. These “selfish” biosynthetic programs are required for cancer cell survival, proliferation, metastasis, and suppression of antitumor immune responses, ultimately leading to tumor progression 18. Metabolic reprogramming of cancer is mainly driven by diverse oncogenic pathways and oncogenes (e.g., PI3K/Akt/mTOR pathway, hypoxia-inducible factor, Ras, c-Myc) 18, 19. However, the role of the Hh-GLI1 signaling pathway in cancer metabolism has not been well studied. In particular, the Warburg phenotype has been shown to have a stronger association with TNBC than with luminal breast cancers 20. Therefore, understanding the unknown relationship between the Hh-GLI1 signaling pathway and TNBC metabolic reprogramming may reveal a new approach to treating TNBC.

This study aimed to investigate the oncogenic role of GLI1 in TNBC, particularly its role in glycolytic metabolism. We analyzed GLI1 expression in TNBC and demonstrated a correlation between GLI1 expression and TNBC cell progression. GLI1 knockdown decreased glycolytic rate and lactate production. Moreover, GLI1 downregulation resulted in reduced cell viability and induced autophagy via the activation of adenosine monophosphate-activated protein kinase. Our results indicate that the Hh-GLI1 signaling pathway regulates proliferation and glycolytic metabolism in cancer, which can provide novel perspectives for TNBC treatment.

## Materials and Methods

### Reagents and antibodies

Roswell Park Memorial Institute-1640 (RPMI-1640) medium and fetal bovine serum (FBS) were purchased from WelGENE (Seoul, South Korea). Antibiotic-antimycotic, Dulbecco's phosphate-buffered saline, and trypsin were purchased from Gibco Life Technologies (Carlsbad, CA, USA). Opti-MEM, Lipofectamine RNAiMAX, and Pierce bicinchoninic acid (BCA) protein assay kits were obtained from Thermo Fisher Scientific (Waltham, MA, USA). Propidium iodide (PI) solution; 4, 6-diamidino-2-phenylindole dihydrochloride; acridine orange hydrochloride; water for high-performance liquid chromatography (HPLC); and thiamine were obtained from Sigma-Aldrich (St. Louis, MO, USA). Liquid diaminobenzidine (DAB)-substrate chromogen system, Mayer's hematoxylin, and antibody diluent were purchased from Dako (Agilent, Santa Clara, CA, USA). The PRO-PREPTM protein extraction solution was purchased from Intron Biotechnology (Daejeon, Korea). Immobilon Forte Western horseradish peroxidase (HRP) substrate and polyvinylidene difluoride (PVDF) membranes were purchased from Millipore (Burlington, MA, USA). HPLC-grade acetonitrile was purchased from JT Baker/Avantor (Center Valley, PA, USA). DL-lactic acid was obtained from the Tokyo Chemical Industry (Tokyo, Japan). Antibodies against cyclin-dependent kinase (CDK)-4 (#12790), pyruvate kinase M2 (PKM2) (#4053), lactate dehydrogenase A (LDHA) (#3582), autophagy-related protein (Atg)-12 (#4180), p-AMPK (#2531), and p-ADD (#3661) were purchased from Cell Signaling Technology (Danvers, MA, USA). Cyclin D1 (sc-718), p21 (sc-6246), p-Rb (sc-16670), Rb (sc-50), Bax (sc-7480), Bcl-2 (sc-7382), glucose transporter 1 (Glut1; sc-7903), monocarboxylate transporter 4 (MCT4; sc-376465), and β-actin (sc-47778) antibodies were purchased from Santa Cruz Biotechnology (Dallas, TX, USA). Ptch (H00005727-A01) antibody was purchased from Thermofisher (Waltham, MA, USA). Rabbit polyclonal antibodies against GLI1 (ab217326) and LC3B (ab51520) were purchased from Abcam (Cambridge, UK). Beclin 1 antibody (NB500-249) and secondary antibodies were obtained from Novus Biologicals (Centennial, CO, USA).

### Cell culture

The human breast adenocarcinoma cell lines MDA-MB-231 and MCF-7 were purchased from the American Type Culture Collection (Manassas, VA, USA). MDA-MB-231 cells were maintained in RPMI-1640 medium containing 10% FBS and 1× antibiotic-antimycotic solution at 37°C and 5% CO_2_.

### Tissue microarray construction

Tissue microarray (TMA) slides from 9 normal tissues and 74 breast cancer tissues were purchased from US Biomax Inc. (Rockville, MD, USA). Detailed information about on TMA samples is provided in Table 1.

### Immunohistochemistry (IHC)

Immunohistochemical analysis of human breast cancer tissues in TMA was performed according to the avidin-biotinylated-HRP complex (ABC) method using an *Elite* ABC kit (Vector Laboratories, Burlingame, CA, USA). The TMA slides were transferred to a xylene chamber and dipped in a graded ethanol series. After antigen retrieval in 10 mM sodium citrate buffer (pH 6.0) for 15 min, the slides were transferred to 3% hydrogen peroxide in methanol to quench the endogenous peroxidase activity. To block non-specific binding sites, slides were incubated in diluted normal goat serum at 24°C for 30 min. The slides were then incubated overnight at 4°C with anti-GLI1 (ab217326, 1:1000). The slides were washed twice with TBS and incubated with biotinylated goat anti-rabbit IgG (1:200) for 30 min at 24°C. After rinsing the slides in TBS, they were incubated with ABC reagent for 30 min. Color development was achieved by applying DAB solution for 2 min. After washing in distilled water, the slides were counterstained with hematoxylin, dehydrated with ethanol and xylene, and coverslipped using a mounting solution. Images were captured using a Zeiss Axiovert 200 microscope (Zeiss, Germany) equipped with a PAXCAM microscope camera.

### Small interfering RNA (siRNA) transfection

MDA-MB-231 cells were transfected with siRNA using Lipofectamine RNAiMAX in Opti-MEM media and the following siRNAs were used at a final concentration of 20 nM: GLI1 siRNA-1 (hs.Ri.GLI1.13.1, Integrated DNA Technologies (IDT) Inc., Coralville, IA), GLI1 siRNA-2 (hs.Ri.GLI1.13.2, IDT), GLI1 siRNA-3 (hs.Ri.GLI1.13.3, IDT), and control siRNA (Negative Control DsiRNA, 51-01-14-03). Downregulation of GLI1 expression was measured by quantitative polymerase chain reaction and immunoblotting analysis after transfection. Transfection efficiency was determined using TYE 563-labeled transfection control DsiRNA (IDT). The cells were transfected with TYE 563-labeled control siRNA (final concentration of 20 nM). After 24 h of incubation, cells were trypsinized and harvested via centrifugation. The siRNA transfection efficiency was observed using a confocal microscope and determined using flow cytometry (ACEA Bioscience, Inc. San Diego, CA, USA).

### Cell viability assay

The IncuCyte ZOOM live cell imaging system (Essen BioScience, Ann Arbor, MI, USA) was used to determine the viability of the breast cancer cells. MCF-7 and MDA-MB-231 cells were seeded at a density of 5000 cells/well in a 96-well plate and transfected with control or GLI1 siRNA at a concentration of 20 nM. The culture medium was aspirated and replaced with fresh medium 6 h after transfection. MDA-MB-231 cells were treated with the GLI1 inhibitor GANT61 (5 - 40 μM) for 24 and 48 h. Cell viability was measured using an MTT assay.

### Western blotting analysis

The harvested cells were washed with cold PBS and lysed in a PRO-PREP protein extraction solution. Supernatants were collected after centrifugation at 12,000 x g for 10 min at 4°C. Protein concentrations of the cell lysates were determined using the Pierce BCA Protein Assay Kit. The proteins (20 μg) were then separated using sodium dodecyl sulfate-polyacrylamide gel electrophoresis (SDS-PAGE) and transferred to PVDF membranes. Membranes were blocked and incubated with primary antibodies at 4 °C overnight. After washing with Tris-buffered saline containing 0.2% Tween-20 (TBS-T), membranes were incubated with HRP-conjugated anti-mouse or anti-rabbit antibodies. Blots were developed using Immobilon Forte Western HRP substrate and detected using a WSE-6100 LuminoGraph chemiluminescence imaging system (ATTO, Tokyo, Japan).

### Quantitative reverse transcription-polymerase chain reaction (qRT-PCR) analysis

Total RNA was extracted from cells using Qiazol Lysis Reagent (Qiagen), according to the manufacturer's protocol. After RNA quantification, cDNA was synthesized via reverse transcription using Reverse Transcription Master Premix (Elpis Biotech). qRT-PCR was performed on a LightCycler 96 Real-Time PCR system (Roche) using the FastStart Essential DNA Green Master (Roche). The 2^-ΔΔCt^ values were calculated to obtain the relative fold-expression levels.

### Cell cycle analysis using flow cytometry

MDA-MB-231 cells were seeded at a density of 20 × 10^4^ cells/well in a 6-well plate and transfected with the control or GLI1 siRNA at 20 nM. The culture medium was aspirated and replaced with fresh medium 6 h after transfection. At 48 h after transfection, the cells were harvested and fixed with 70% ethanol for 24 h at -20 °C. The cells were washed with PBS and resuspended in PI staining solution (1 μg/mL RNase A and 10 μg/mL PI in PBS) for 30 min at 37°C. The stained cells were analyzed using a NovoCyte flow cytometer (ACEA Bioscience, Inc. San Diego, CA, USA). The percentage of cells in the G0/G1, S, and G2/M phases was determined using the NovoExpress software (ACEA Bioscience, Inc.).

### Apoptosis analysis using flow cytometry

An annexin V-FITC apoptosis detection kit (BD Biosciences, San Jose, CA, USA) was used to assess apoptotic cell death. MDA-MB-231 cells were seeded at a density of 20 × 10^4^ cells/well in a 6-well plate and transfected with the control or GLI1 siRNA at 20 nM. The culture medium was aspirated and replaced with fresh medium 6 h after transfection. At 48 h after transfection, the cells were harvested and fixed with 70% ethanol for 24 h at -20°C. After centrifugation, cells (1 × 10^6^ cells/mL) were resuspended in 1× binding buffer. Then, 100 μL of the solution was transferred to a 5-mL culture tube to which 5 μL of annexin V-FITC and 5 μL of PI were added, and the tubes were incubated for 30 min at 37°C. The stained cells were analyzed using a NovoCyte flow cytometer (ACEA Bioscience, Inc. San Diego, CA, USA). The percentage of cells in the G0/G1, S, and G2/M phases was determined using the NovoExpress software (ACEA Bioscience, Inc.).

### Seahorse XF analysis of glycolytic rate

MDA-MB-231 cells were seeded into Seahorse XF96 cell culture microplates at a density of 5000 cells/well, and transfected with 20 nM siRNA. At 48 h post-transfection, the glycolytic rate was measured using a Seahorse XFe96 analyzer (Agilent, Santa Clara, CA, USA). On the day of the assay, the medium was changed to Seahorse XF RPMI medium (pH 7.4), supplemented with 2000 mg/L D-glucose and 300 mg/L L-glutamine. The extracellular acidification rate (ECAR) was recorded at 6 min intervals at baseline, followed by treatment with 0.5 μM rotenone/antimycin A and 50 mM 2-deoxy-D-glucose (2-DG) (XF Glycolytic Rate Assay Kit).

### Lactate measurement

MDA-MB-231 cells were seeded at a density of 20 × 10^4^ cells/well in a 6-well plate and transfected with the control or GLI1 siRNA at a concentration of 20 nM. After 48 h, the cell medium was collected and mixed by vortexing with acetonitrile containing the internal standard (IS) thiamine. After centrifugation at 4000 rpm for 5 min, the supernatants were collected and analyzed. Lactate content in the cell media was measured using HPLC. The liquid chromatography (LC) system used in this study comprised a Gilson pump (LC-321 322 350 pump), autosampler (Gilson-234), and UV (UV/VIS-151) detector (Gilson, France). Detection and quantification were performed on a C18 column (Synergi 4 µm Hydro-RP 80 Å, LC Column 250 × 4.6 mm) preceded by a pre-column (Phenomenex, USA). The isocratic mobile phase of water with 0.1% phosphoric acid was used for the analysis (0.8 mL/min flow rate, RT). Lactate concentration was measured using a UV detector at 210 nm.

### Acridine orange staining

MDA-MB-231 cells were seeded in 35 mm confocal plates, and after 24 h, the cells were transfected with the control or GLI1 siRNA. The culture medium was aspirated and replaced with fresh medium 6 h after transfection. At 48 h post-transfection, cells were stained with 1 μg/mL acridine orange for 15 min at RT. After washing twice with cold PBS, the cells were examined under a K1-Fluo confocal microscope (Nanoscope Systems, Daejeon, Korea).

### Statistical Analysis

Unless otherwise indicated, all data are presented as the mean ± standard deviation (SD) of at least three independent experiments. Statistical analysis was performed using SPSS software (version 21.0; SPSS Inc., Chicago, IL, USA). Statistical analysis was conducted using a two-tailed Student's *t*-test for unpaired samples. *P*<0.05 was considered statistically significant compared with the control.

## Results

### Analysis of GLI1 expression levels in the samples of patients with breast cancer and breast cancer cell lines

**Figure 1:**
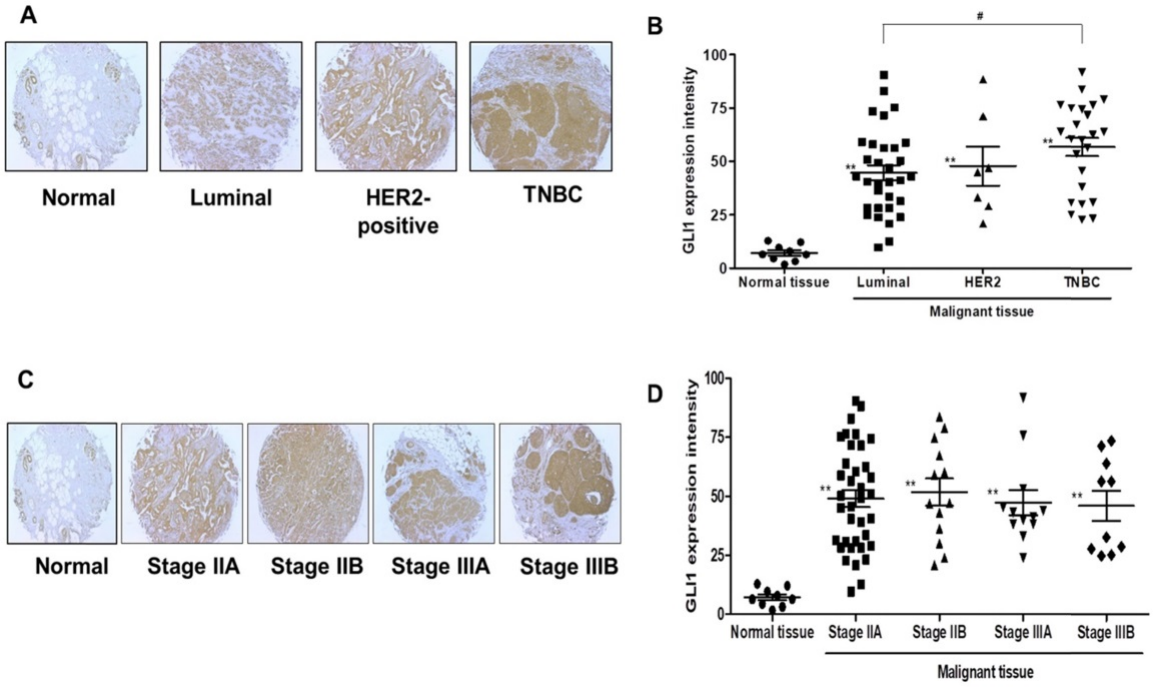
Analysis of glioma-associated oncogene 1 (GLI1) expression in a tissue microarray. (**A and C**) Representative immunohistochemical staining of GLI1 in human normal breast tissue and breast cancer tissues. (**B and D**) Quantitative analysis of GLI1 expression in breast cancer tissues. Data showed that the expression levels of GLI1 are higher in breast cancer tissues than in normal breast tissues. Triple-negative breast cancer (TNBC) samples exhibited higher GLI1 expression levels compared to that in the luminal tumor samples, but GLI1 expression did not correlate with the tumor stage. Data are shown as the means ± standard error of the mean (SEM). **p < 0.01 as compared with the normal tissues; #*p* < 0.05 as compared with the luminal tumors.

**Figure 2:**
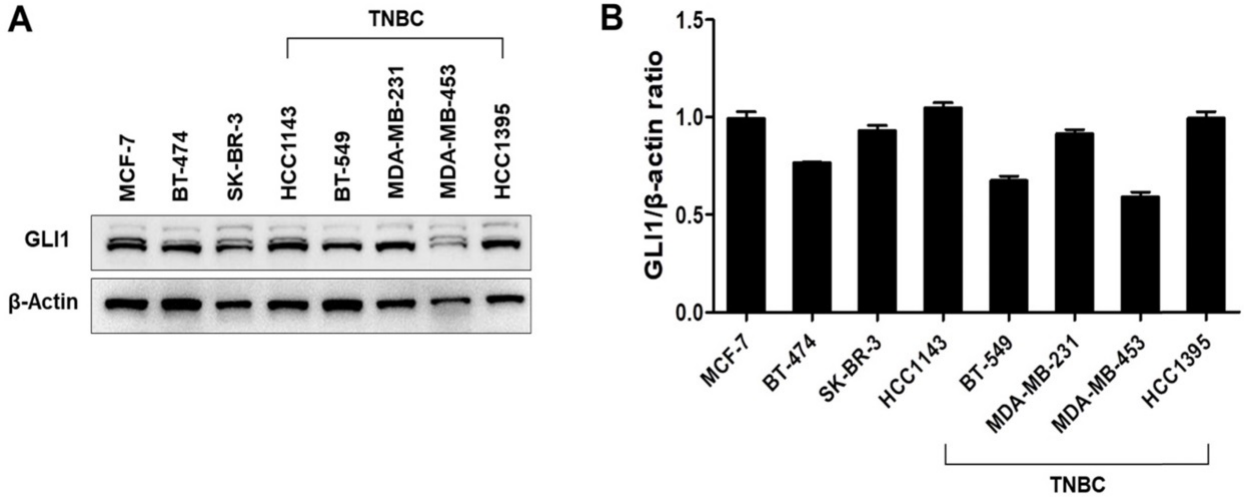
Expression levels of GLI1 in breast cancer cell lines. (**A**) Representative western blot of GLI1 expression levels in a panel of established breast cancer cell lines. (**B**) Representative bar graph showing the quantified expression levels of GLI1 to β-actin in various breast cancer cell lines. Data are shown as the means ± SEM of triplicate experiments.

GLI1 expression was examined in 74 breast cancer tissues and compared with the expression in nine normal breast tissues based on molecular subtypes and tumor stages. As shown in Figure 1A, GLI1 protein expression was localized in both the nucleus and cytoplasm and was significantly higher in breast cancer tissues than in normal tissues. Compared to the molecular subtypes, GLI1 was overexpressed to a greater extent in TNBC (mean intensity ± standard error of the mean (SEM): 56.81 ± 4.297) than in luminal tumors (mean intensity ± SEM: 44.64 ± 3.528). However, the expression of GLI1 was not correlated with tumor stage (Figure 1D). The clinicopathological features of all tissues are summarized in Table 1. GLI1 expression has also been analyzed in various breast cancer cell lines. GLI1 was highly expressed in all the breast cancer cell lines, with the lowest expression observed in MDA-MB-453 cells (Figure 2).

### Analysis of siRNA transfection efficiency and downregulation of GLI1

**Figure 3:**
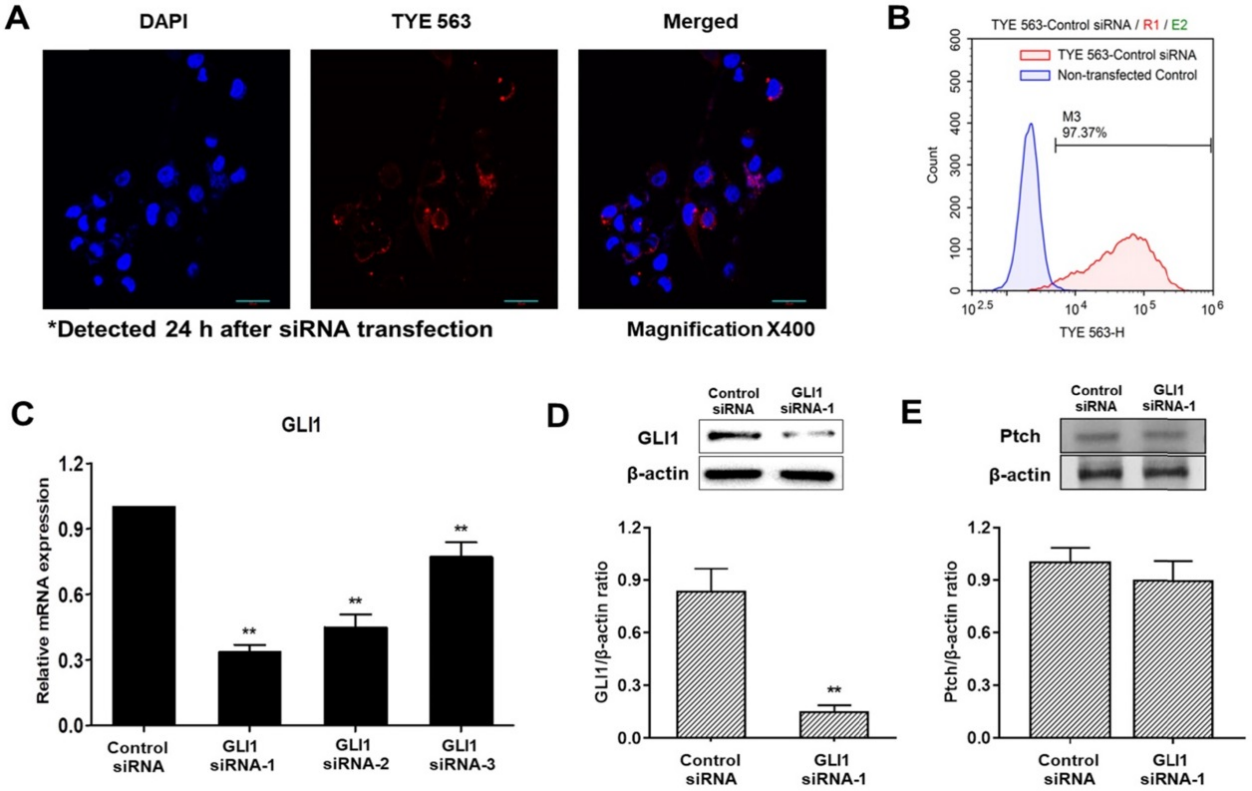
Detection of the transfection efficiency of small interfering RNA (siRNA) and GLI1 expression. (**A**) Confocal images showing the uptake of siRNA in MDA-MB-231 cells (magnification × 400). First, 20 nM of TYE 563-labeled-control siRNA was transfected into the cells. The nuclei were stained with 4, 6-diamidino-2-phenylindole dihydrochloride. siRNA entered the cells and was primarily distributed in the cytoplasm. (**B**) Flow cytometry analysis of siRNA transfection efficiency in MDA-MB-231 cells. The siRNA transfection efficiency was approximately 97%. (**C**) Quantitative reverse transcription-polymerase chain reaction (qRT-PCR) analysis of MDA-MB-231 cells transfected with the indicated GLI1 siRNAs. GLI1 siRNA-1 considerably reduced GLI1 expression as compared with that in the other groups. Data are shown as the means ± SEM of triplicate experiments. (**D**) Expression levels of GLI1 protein after GLI1 siRNA-1 transfection as analyzed by western blotting. (**E**) Expression levels of Pitch protein after GLI1 siRNA-1 transfection as analyzed by western blotting. Data are shown as the means ± SEM of triplicate experiments. **p < 0.01 compared with control the siRNA group.

To determine transfection efficiency, a TYE 563-conjugated transfection control siRNA was used. The transfection control DsiRNA is a non-targeting DsiRNA that does not interact with any sequence in the human transcriptome. Confocal fluorescence images of cells transfected with control DsiRNA are shown in Figure 3A. The red fluorescence signal of the control DsiRNA was primarily distributed in the cytoplasm. Histograms of non-transfected cells (blue) and TYE 563-labeled control DsiRNA-transfected cells (red) obtained using flow cytometry are shown in Figure 3B. The siRNA transfection efficiency in MDA-MB-231 cells was 97%, sufficient to precede the experiments. Quantitative PCR was used to compare the inhibitory effects of three pairs of siRNAs against GLI1. As shown in Figure 3C, GLI1 siRNA-1 exhibited the most potent inhibition of GLI1 mRNA expression. Therefore, GLI1 siRNA-1 was selected for the subsequent experiments. Knockdown of GLI1 expression mediated by GLI1 siRNA-1 was confirmed by western blot analysis (Figure 3D). The relative protein levels of GLI1 were also markedly higher in MDA-MB-231 cells. However, Ptch expression did not change in the GLI1 siRNA-transfected MDA-MB-231 cells (Figure 3E). Therefore, the components of the Shh-GLI1 signaling pathway are overexpressed in the human breast cancer cell line MDA-MB-231. This suggests that Ptch activates shh-GLI1 signaling in MDA-MB-231 cells.

### GLI1 promotes TNBC proliferation and cell cycle progression

**Figure 4:**
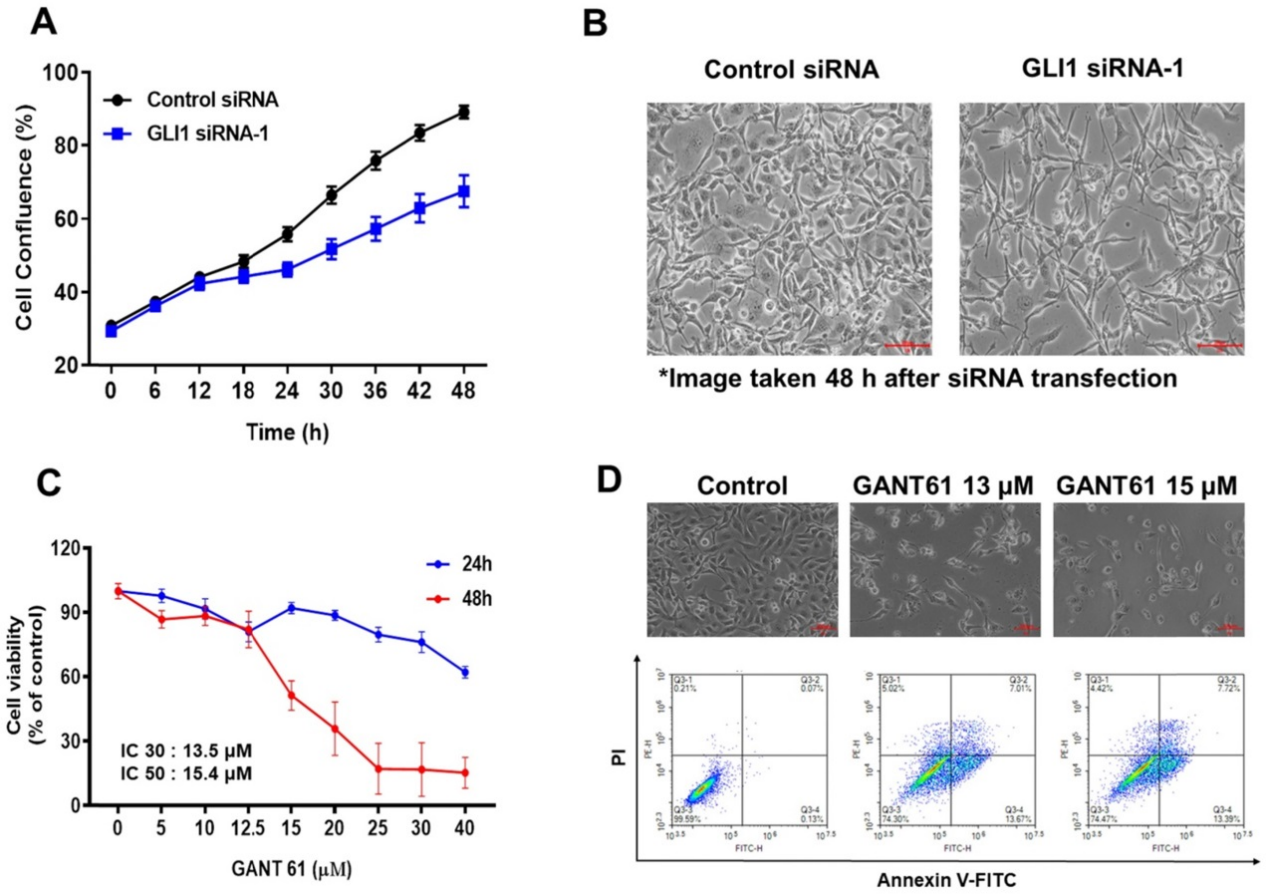
Effect of GLI1 knockdown on the cell viability in MDA-MB-231 cells. (**A**) MDA-MB-231 cells transfected with the control or GLI1 siRNA were monitored for their proliferation using IncuCyteZOOM live cell imaging system. At a concentration of 20 nM, GLI1 siRNA significantly reduced the proliferation of MDA-MB-231 cells, compared with that in the control siRNA-transfected group. (**B**) Morphology of MDA-MB-231 cells transfected with GLI1. Magnification ×100. Morphological changes were prominent after GLI1 knockdown. (**C**) Effect of GLI1 knockdown by GANT61 on the cell viability in MDA-MB-231 cells. MDA-MB-231 cells treated with GANT61 for 24 and 48 h. The cell viability was monitored using IncuCyteZOOM live cell imaging system. (**D**) Morphology of MDA-MB-231 cells treated with GANT61 and apoptosis was determined by the Annexin-V/PI assay in MDA-MB-231 cells treated with GANT61 for 48 h.

To explore the effect of GLI1 knockdown on TNBC, MDA-MB-231 cells were imaged every 6 h using IncuCyte ZOOM (Satorius), and the results were analyzed using the IncuCyte ZOOM software. Transfection with GLI1 siRNA-1 reduced the viability of MDA-MB-231 cells compared with that in the negative control siRNA group (Figure 4A). Furthermore, downregulation of GLI1 not only reduced viability but also induced remarkable morphological changes in MDA-MB-231 cells (Figure 4B). To confirm that GLI1 inhibition can affect the cytotoxicity of breast cancer cells, we compared the cytotoxicity of MDA-MB-231 cells treated with a specific GLI1 inhibitor, GANT61. As shown in Figures 4C and 4D, GANT61 significantly reduced the viability of MDA-MB-231 cells in a concentration-dependent manner. In addition, the apoptotic cell population was markedly increased in GANT61-treated MDA-MB-231 cells compared to the control. We evaluated the effects of the direct silencing of GLI1 expression on cell viability by transfecting GLI1 siRNA in MCF-7 cells. Furthermore, a significant decrease in cell proliferation was observed in GLI1 siRNA-treated MCF-7 cells, as assessed by the MTT assay (Supplementary Figure 1).

**Figure 5:**
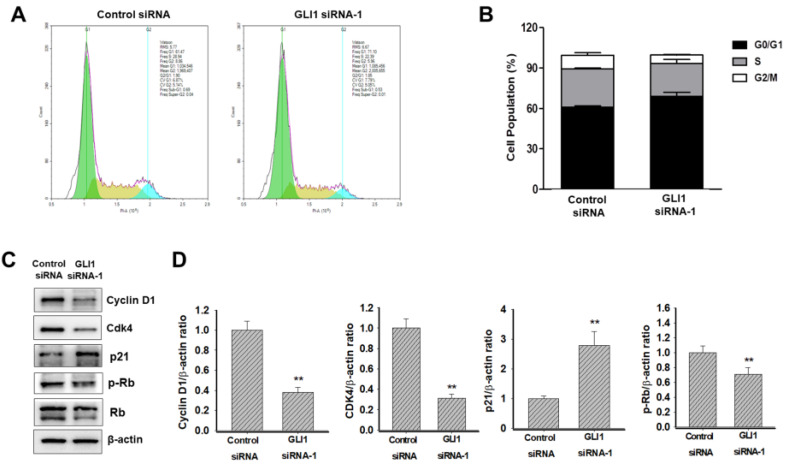
Effect of GLI1 knockdown on the cell cycle progression in MDA-MB-231 cells. (**A**) Cell cycle analysis using flow cytometry in MDA-MB-231 cells transfected with the control or GLI1 siRNA. (**B**) Quantitative distribution of cell cycle in siRNA-transfected MDA-MB-231 cells. GLI1 downregulation resulted in G1 arrest. Data are shown as the means ± standard deviation (SD) of triplicate experiments. (**C**) Expression levels of cell cycle regulatory proteins analyzed by western blotting. (**D**) Intensities of the bands were measured and depicted in the bar graph as the ratio of the expression of the protein to that of β-actin. **p < 0.01 compared with the control siRNA-transfected cells.

Next, we determined whether GLI1 regulated the cell cycle in MDA-MB-231 cells. Flow cytometry analysis of DNA content established that the knockdown of GLI1 led to G1 cell cycle arrest (Figure 5A). The relative distribution of cells in different phases of the cell cycle is shown in Figure 5B. The progression of the cell cycle from the G1 to S phase is controlled by the activity of cyclins (types D and E) in conjunction with G1 phase cyclin-dependent kinases (cdk2, cdk4, and cdk6). These cyclin-cdk complexes induce the phosphorylation of Rb protein, resulting in the release of transcription factor E2F, which is required for the transcription of S-phase genes 21. The activities of cyclin-cdk complexes are tightly regulated by inhibitor proteins such as p27 and p21 22. To understand the mechanism of G1 cell cycle arrest exerted by GLI1 knockdown, a western blot analysis of cell cycle regulatory proteins was performed. As shown in Figure 5C, significant reductions in the expression of cyclin D1 and cdk4 were caused by GLI1 knockdown. Furthermore, increased phosphorylation of p21 and decreased phosphorylation of Rb were detected after the downregulation of GLI1 (Figure 5D). These data suggested that GLI1 knockdown inhibits cell proliferation via p21-mediated G1 arrest.

### GLI1 silencing induces TNBC apoptosis

**Figure 6:**
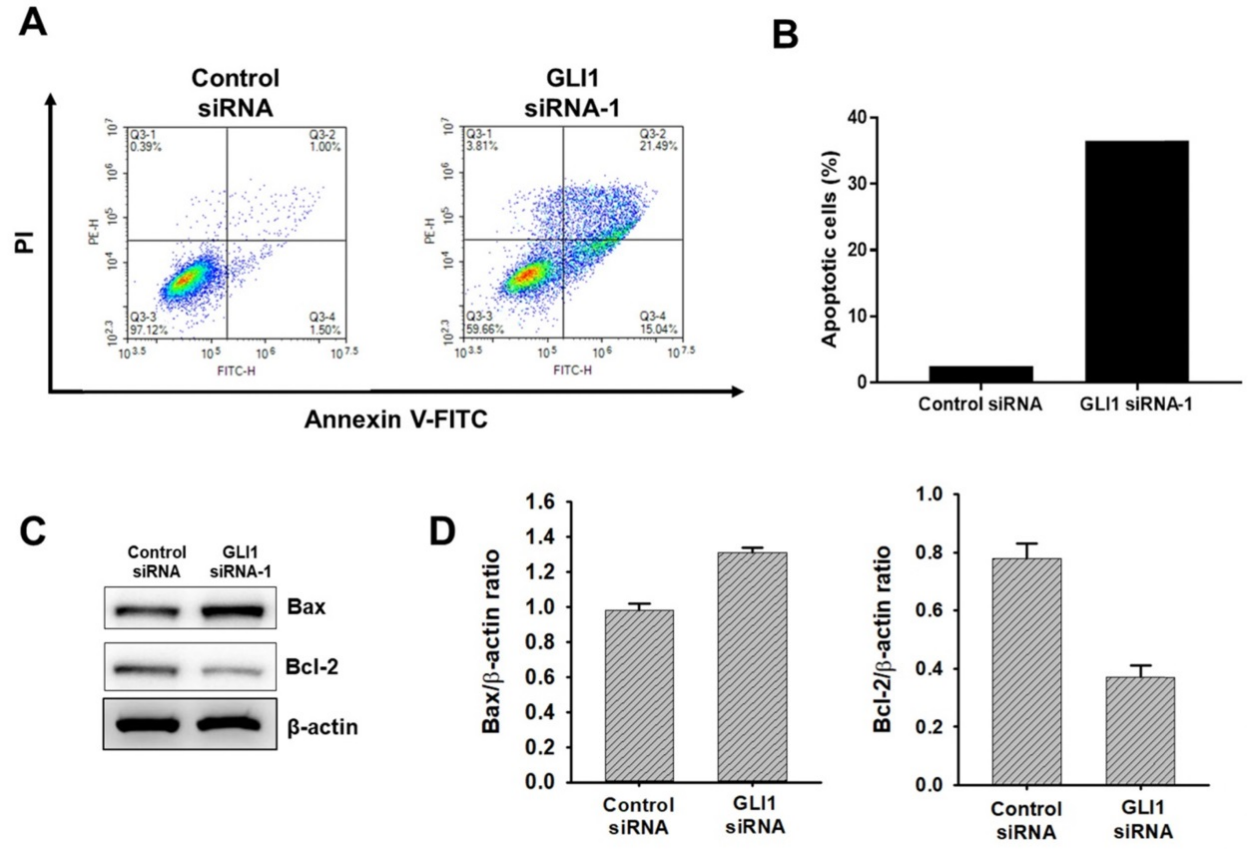
Effect of GLI1 knockdown on the apoptotic cell death in MDA-MB-231 cells. (**A**) GLI1 siRNA knockdown in MDA-MB-231 cells results in increased apoptosis and decreased levels of anti-apoptotic proteins. (**A**) Apoptosis was determined by the Annexin-V/PI assay for MDA-MB-231 cells transfected with GLI1 siRNA or control siRNA for 48 h. (**B**) Quantitative distribution of apoptotic cell population in GLI1 siRNA-transfected MDA-MB-231 cells (**C**) Immunoblots of Bax and Bcl-2 protein expression in MDA-MB-231 cells transfected with GLI1 siRNA or control siRNA for 48 h. The blots were stripped and reprobed for *β*-actin as an internal control for equal loading. (**D**) Data indicate values performed in triplicate and the fold difference relative to *β*-actin. Data are shown as the means ± standard deviation (SD) of triplicate experiments.

To determine if the observed decrease in proliferation seen with GLI1 siRNA-transfected cells due to an increase in apoptosis, Annexin-V staining was performed. As shown in Figure 6A and 6B, GLI1 siRNA treated MDA-MB-231 cells had a 3.2-fold increase in Annexin-V-positive cells (58%) over the control siRNA-transfected cells (18%). To further understand the mechanism of the observed cell death, immunoblot analysis was conducted to examine the effect of GLI1 knockdown on the expression of key apoptotic signalling proteins. Immunoblot analysis (Figure 6C and 6D) shows that at 48 h, GLI1 siRNA drastically decreased the expression of Bcl-2 compared to control siRNA. This corresponded with a significant increase in the expression of Bax, an anti-apoptotic protein that is a key determinant of apoptotic regulation.

### GLI1 regulates glucose metabolism in TNBC cells

**Figure 7:**
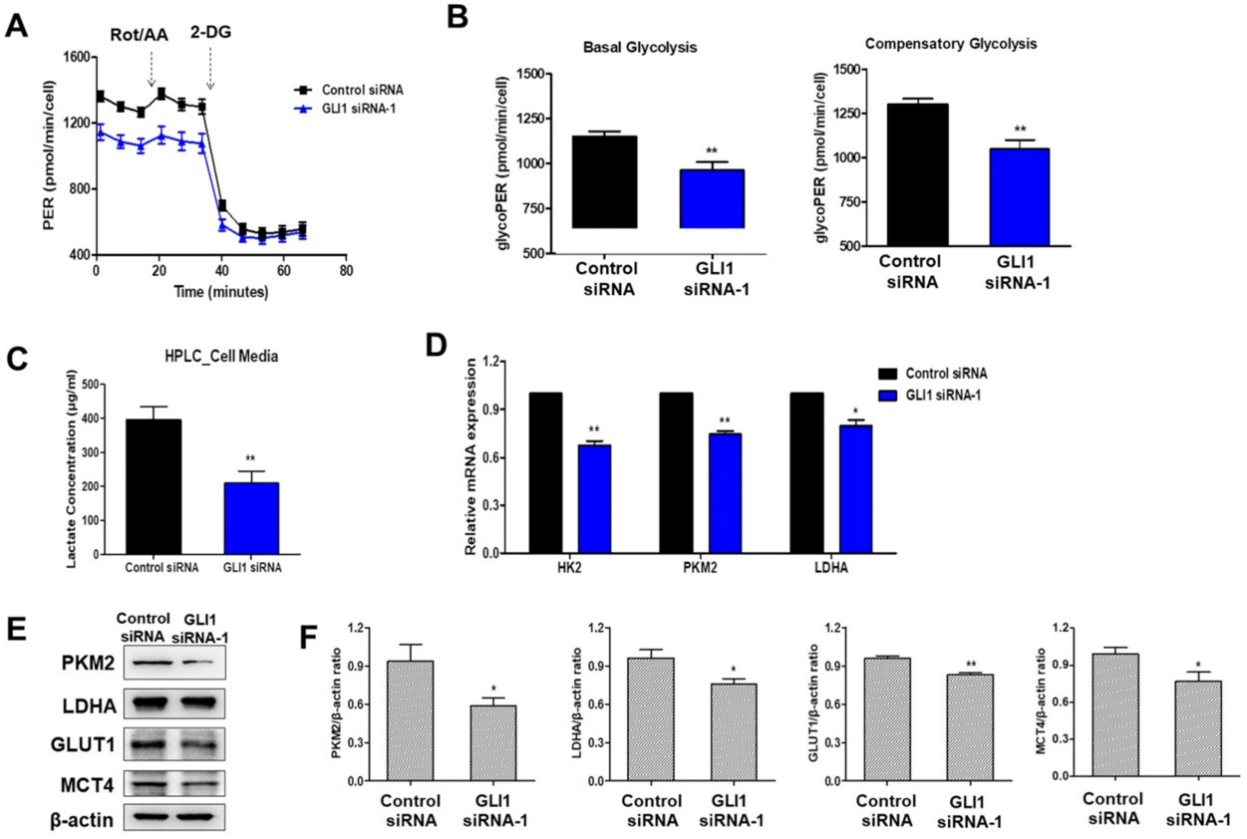
Downregulation of GLI1 attenuates glucose metabolism in MDA-MB-231 cells. (**A**) Glycolytic rate of MDA-MB-231 cells after siRNA transfection was measured using the XF Glycolytic Rate Assay Kit. (**B**) Both basal and compensatory glycolysis decreased after GLI1 knockdown. (**C**) Quantitative levels of lactate in cell media. GLI1 siRNA significantly reduced the lactate levels in the media of MDA-MB-231 cells. **p < 0.01 as compared with the control siRNA-transfected cells. (**D**) Expression patterns of glucose metabolism-related proteins after GLI1 knockdown by siRNA. qRT-PCR analysis of key glycolytic enzymes in siRNA-transfected MDA-MB-231 cells. (**E**) Western blot analysis of glucose metabolism-related proteins in MDA-MB-231 cells. (**F**) Intensities of the bands were measured and depicted in the bar graph as the ratio of the expression of the protein to that of β-actin. *p < 0.05, **p < 0.01 compared with the control siRNA-transfected cells.

Cancer cells exploit the distinct glucose metabolism of aerobic glycolysis (Warburg effect) for rapid energy production 17. We investigated whether the Hh-GLI1 signaling pathway is involved in the modulation of glucose metabolism in TNBC cells. Therefore, we measured the glycolytic rates of siRNA transfected-MDA-MB-231 cells using the Seahorse XF glycolytic rate assay. GLI1 knockdown in MDA-MB-231 cells resulted in a decrease in basal glycolysis and compensatory glycolysis, indicating that aerobic glycolysis rates were decreased compared to those in the control group (Figures 7A and 7B). To determine the effect of GLI1 silencing on the generation of lactate, the major metabolite in cancer cells, we quantitatively analyzed lactate levels in the media of MDA-MB-231 cells using HPLC. Knockdown of GLI1 expression considerably reduced the extracellular lactate concentration (Figure 7C). Moreover, GLI1 knockdown in MDA-MB-231 cells resulted in downregulation of the mRNA levels of key glycolytic enzymes, hexokinase 2 (HK2), PKM2, and LDHA (Figure 7D), which contributed to the reduction in glycolysis rate. Furthermore, we confirmed protein expression levels using western blot analysis in cell lysates of GLI1 siRNA-transfected MDA-MB-231 cells. As shown in Figures 7E and 7F, GLI1 downregulation inhibited the expression of GLUT1 and MCT4. Downregulation of these transporters may explain the decreased glycolysis rate and lowered extracellular lactate concentration.

### GLI1 knockdown induces autophagy in TNBC cells

**Figure 8:**
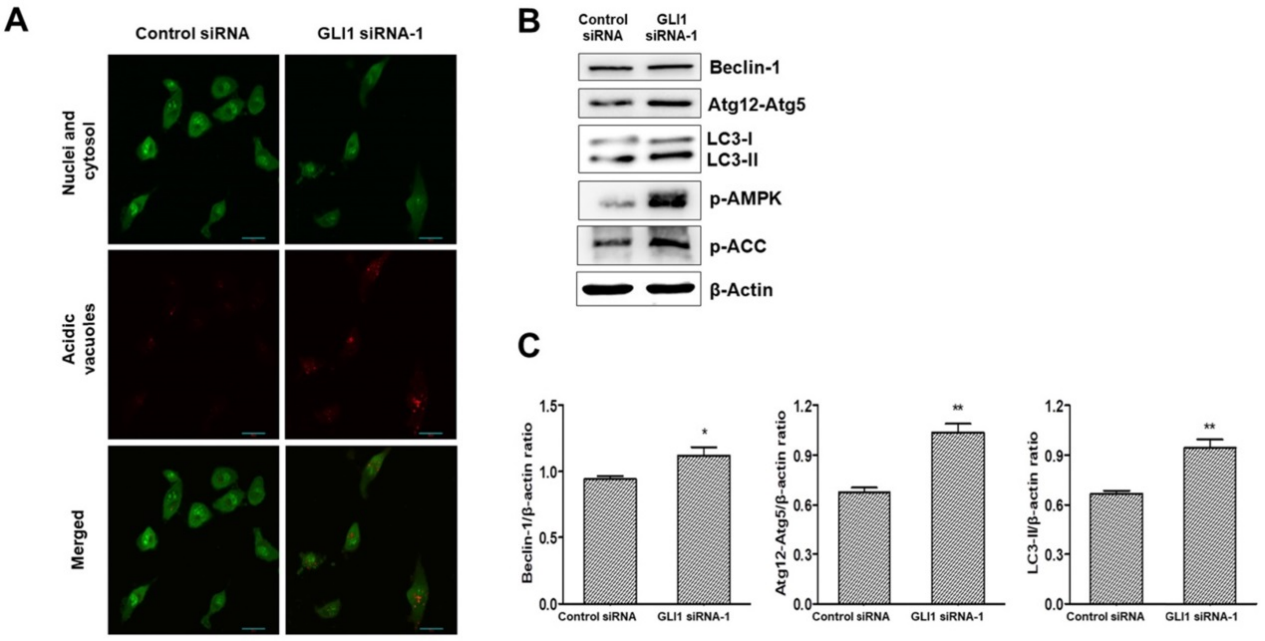
Analysis of autophagy induction after GLI1 silencing. (**A**) Acidic vacuoles were observed under a confocal microscope at 400× magnification. After staining with acridine orange (1 μg/mL), fluorescent green staining of nuclei and cytoplasm, and fluorescent bright red staining of the autophagic vacuoles were observed. (**B**) Immunoblots of autophagy-related proteins in MDA-MB-231 cells. (**C**) Bar graphs indicating the relative expression levels of proteins compared to that of β-actin. *p < 0.05, **p < 0.01 compared to the control siRNA group.

It is debatable whether Hh signaling inhibits or upregulates autophagy in cancer 23. To investigate whether GLI1 plays a role in regulating autophagy, we performed acridine orange staining in siRNA-transfected MDA-MB-231 cells. As depicted in Figure 8A, GLI1 knockdown considerably induced autophagic vacuole formation (bright red fluorescence) compared with that in the control group. LC3-I is converted to LC3-Ⅱ, which is a protein marker reliably associated with completed autophagosomes. Upregulation of LC3-Ⅱ was analyzed using western blot analysis. As shown in Figure 8B, the induction of autophagy was further confirmed through the analysis of other autophagy-related proteins (Beclin-1 and Atg12-Atg5 complex). AMPK is the primary regulator of autophagy. AMPK is known to increase autophagy through diverse direct or indirect regulations (e.g., activation of the ULK1 complex and Class III PI3K complex and phosphorylation of ATG9) 24. We examined the effect of GLI1 knockdown on AMPK activation by measuring the phosphorylation status of AMPK and its target substrate acetyl-CoA carboxylase, using western blot analysis (Figure 8C). These results suggest that autophagy induction after GLI1 knockdown may be mediated by AMPK activation.

## Discussion

TNBC is an aggressive subtype of breast cancer characterized by tumors that do not express the ER, PR, or HER-2 genes, making it unresponsive to endocrine therapy and HER2-targeted treatment 25, 26. Considering the limited treatment options and aggressive phenotype of TNBC, it is crucial to improve our understanding of TNBC features and discover potential therapeutic targets to aid in the development of effective therapies. The Hh-GLI1 signaling pathway is usually turned off in normal cells; however, abnormal activation has been reported in several cancers 27, making it an attractive therapeutic target. In the present study, we investigated the role of the Hh-GLI1 signaling pathway in cancer proliferation and glucose metabolism in TNBC.

In previous studies, high GLI1 expression has been observed in various malignant cancers 28, 29. In this study, we compared the expression of GLI1 in normal and breast cancer tissues. When classified into molecular subtypes (luminal, HER2-enriched, and TNBC), TNBC samples showed higher GLI1 expression than the luminal tumor samples. This finding is in line with that of a previous study that reported higher GLI1 expression in TNBC and HER2-enriched breast cancer 30. High GLI1 expression has been observed in various breast cancer cell lines. These results suggested that GLI1 may play a role in breast tumorigenesis, especially in TNBC.

To investigate the functional role of the Hh-GLI1 signaling pathway in TNBC, we used siRNA-mediated GLI1 knockdown in MDA-MB-231 cells. GLI1 knockdown significantly decreased MDA-MB-231 cell proliferation in a time-dependent manner, suggesting a correlation between GLI1 overexpression and TNBC survival. Previous studies have reported a close relationship between the Hh signaling pathway and cell cycle regulators. Through gene expression profiling, GLI1 was reported to regulate cyclin D2 expression by directly binding to its promoter 31. In human epithelial cells, p21-induced cell cycle arrest is suppressed by Shh 32. In this study, GLI1 knockdown resulted in G1 cell cycle arrest, and subsequent western blot analysis showed that it was mediated by the activation of p21, a potent CDK inhibitor. In previous data, GLI1 mediated HH pathway can induce apoptosis in various cancer cells 33. Hence, GLI1 can be a future diagnostic and prognostic marker as well as a potent therapeutic target in breast cancer. To validate the role of GLI1 expression in MDA-MB-231 cell growth, we investigated the direct inhibition of GLI1 expression by RNA interference. We observed an increased proportion of early apoptotic and late apoptotic cells 48 h after GLI1 knockdown. The decrease in cell proliferation and increase in apoptosis in MDA-MB-231 cells treated with GLI1 siRNA corresponded to the decrease in the levels of the most potent anti-apoptotic protein Bcl-2.

Metabolic reprogramming of cancer cells is emerging as an attractive therapeutic target, and there has been growing interest in the unknown mechanisms underlying the rewired metabolic network. However, only a few studies have explored the involvement of Hh-GLI1 signaling and metabolic reprogramming in cancer; the reports have been contradictory. In medulloblastoma, Shh activation induces transcription of HK2 and PKM2, causing a robust increase in glycolysis. This process is mediated by the canonical activation of GLI transcription factors 34. However, Glabrescione B (GlaB, a GLI1 inhibitor) increases glucose uptake and lactate excretion, exacerbating the Warburg effect 35. In a breast cancer study, Shh stimulated glycolytic metabolism by regulating 6-phosphofructo-2-kinase/fructose-2,6-biphosphatase 3 (PFKFB3) activation. This regulation is mediated by Smo, but not by GLI1 36.

In this study, we investigated the metabolic profile of MDA-MB-231 cells using a Seahorse XFe96 Analyzer. The Seahorse XFe96 Analyzer directly measures the ECAR and oxygen consumption rate (OCR), which are key indicators of glycolysis and OXPHOS, respectively. The glycolytic proton efflux rate (glycoPER) of cells can be determined by measuring both ECAR and OCR. The assay workflow is as follows. First, basal glycolysis rates were recorded over three measurement periods. After the injection of Rot/AA (inhibitors of the mitochondrial electron transport chain), compensatory glycolysis rates were measured. Finally, injection of 2-DG (an inhibitor of glycolysis) confirmed that the previously measured PER was primarily due to glycolysis. A reduction in the basal and compensatory glycolysis rates was observed after GLI1 knockdown. HPLC analysis showed that GLI1 silencing resulted in a significant decrease in extracellular lactate concentration. Our data indicate a potential association between the Hh-GLI1 signaling pathway and glycolysis in MDA-MB-231 cells.

In cancer cells, there are three committed steps in glycolysis that are catalyzed mainly by HK2, phosphofructokinase 1 (PFK1), and PKM2. Pyruvate, the end product of glycolysis, is converted to lactate in cancer cells. LDHA catalyzes the conversion of pyruvate to lactate 37. The expression levels of HK2, PKM2, and LDHA were decreased after GLI1 siRNA transfection in MDA-MB-231 cells. Moreover, the GLUT1 (glucose transporter) and MCT4 (lactate transporter), which are usually highly expressed primarily in cancer cells, were downregulated by GLI1 knockdown. Thus, it can be concluded that the Hh-GLI1 pathway regulates glycolysis by modulating key glycolytic enzymes. AMPK is a key energy sensor in cancer that links various cellular functions and processes to energy availability. AMPK is activated when the AMP:ATP and/or ADP:ATP ratio increases. Once activated, AMPK maintains energy homeostasis by inhibiting ATP-consuming processes, including glycogen, lipid, or protein biosynthesis, and promoting ATP-conserving processes 38. In the present study, GLI1 knockdown significantly induced AMPK activation. It is assumed that GLI1 siRNA-mediated glycolytic impairment leads to an energy-deficient state that consequently activates AMPK. Basal-like breast cancer MDA-MB-231 cells have been reported to exhibit OXPHOS deficiency compared to luminal-like breast cancer MCF-7 cells, showing a significant decrease in mitochondrial respiration 39. It seems that lowered glycolysis could not have been sufficiently compensated for by OXPHOS in the GLI1 siRNA-transfected MDA-MB-231 cells.

Autophagy is a cellular process in which the catabolism of intracellular organelles generates energy for cell survival. However, prolonged autophagy can lead to non-apoptotic type II programmed cell death 40. Although autophagy regulation is complex, and a variety of signaling cascades and regulatory mechanisms modulate autophagic activity, AMPK is probably the most conserved autophagy inducer throughout evolution 24. To explore autophagy induction after GLI1 knockdown, acridine orange staining was used to detect autophagosome accumulation in MDA-MB-231 cells. Subsequent autophagy-related protein (LC3-Ⅱ, Beclin-1, and Atg12-Atg5 complex) expression also confirmed the induction of autophagy after GLI1 knockdown. The identification of GLI1 as a key transcriptional regulator for cancer cell proliferation and cell death pathway highlights its promise as a therapeutic target. Therefore, GLI1 inhibitors are potentially efficacious against human breast tumors arising via multiple oncogenic mechanisms.

## Conclusions

**Figure 9:**
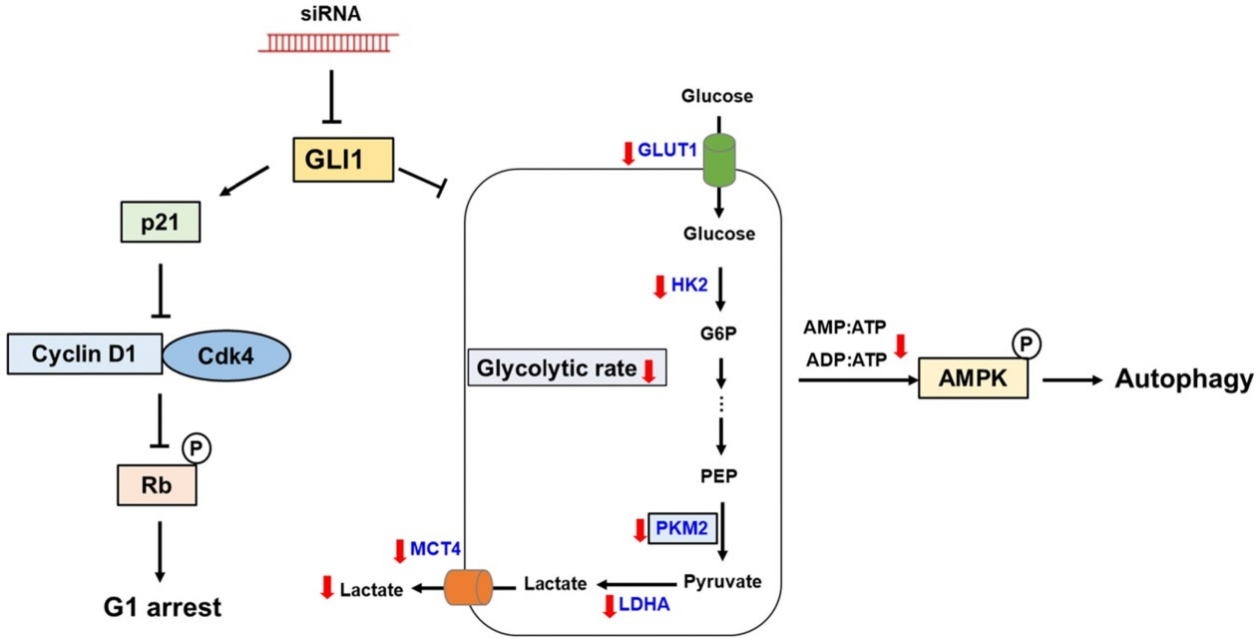
Proposed model links the effects of GLI1 knockdown on TNBC cell survival and glucose metabolism. GLI1 knockdown inhibits TNBC cell proliferation by inducing p21-dependent G1 phase cell cycle arrest. Downregulation of GLI1 impairs glycolytic metabolism via the modulation of key glycolytic enzymes, and consequently activates adenosine monophosphate-activated protein kinase, leading to the induction of autophagy.

In the present study, we demonstrated aberrant overexpression of GLI1 in breast cancer tissues compared to that in normal breast tissues, especially in the TNBC subtype, suggesting a crucial role of the Hh-GLI1 signaling pathway in TNBC tumorigenesis. Our results demonstrate that GLI1 knockdown inhibits TNBC cell proliferation via p21-induced G1 cell cycle arrest and that Hh-GLI1 signaling regulates glycolytic metabolism by modulating glycolytic enzymes (Figure 9). Together, these findings provide a novel perspective for understanding TNBC tumorigenesis and metabolic reprogramming and broaden the knowledge in this field, which can aid in the development of successful treatment strategies for TNBC. Therefore, inhibition of GLI1-target agents may be effective for breast cancer treatment in the future.

## Supplementary Material

Supplementary figures.

## Figures and Tables

**Table 1:** Clinicopathological features of tissues obtained from normal and breast cancer patients.

Variable	No. of samples	Normal tissues (%)		Breast cancer tissues (%)	
		<60	≥60	<60	≥60
Cases (Female)	74 cancer tissues, 9 normal tissues	9 (100%)	-	59 (79.73%)	15 (20.27%)
**Molecular subtypes**					
Luminal		-	-	25 (39.68%)	5 (7.94%)
HER2-enriched		-	-	5 (7.94%)	4 (6.35%)
Triple-negative		-	-	18 (28.57%)	6 (9.52%)
**Tumor stage**					
ⅠA		-	-	-	1 (1.35%)
ⅡA		-	-	30 (40.54%)	7 (9.46%)
ⅡB		-	-	10 (13.51%)	3 (4.05%)
ⅢA		-	-	13 (17.57%)	-
ⅢB		-	-	6 (8.11%)	4 (5.41%)
**Grade**					
1-2		-	-	2 (3.45%)	-
2		-	-	31 (53.45%)	9 (15.52%)
2-3		-	-	2 (3.45%)	-
3		-	-	10 (17.24%)	4 (6.90%)
