# Supplementary material for: Glycolytic Metabolic Remodeling by the Truncate of Glioma-Associated Oncogene Homolog 1 in Triple-Negative Breast Cancer Cells
Source: J Cancer. 2022 Aug 8;13(10):3031–43. doi: 10.7150/jca.72793 (PMC9414023; doi:10.7150/jca.72793)
Supplement: Supplementary file 1 — Supplementary figures. [file jcav13p3031s1.pdf]

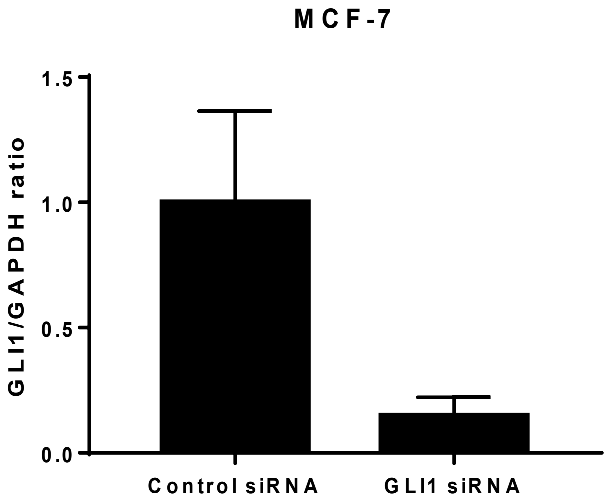

Supplementary Figure 1. Effect of GLI1 knockdown on the cell viability in MCF-7 cells. (**A**) MCF-7 cells transfected with the control or GLI1siRNA were monitored for their proliferation using IncuCyteZOOM live cell imaging system. At a concentration of 20 nM, GLI1 siRNA significantly reduced the proliferation of MCF-7 cells, compared with that in the control siRNA-transfected group.

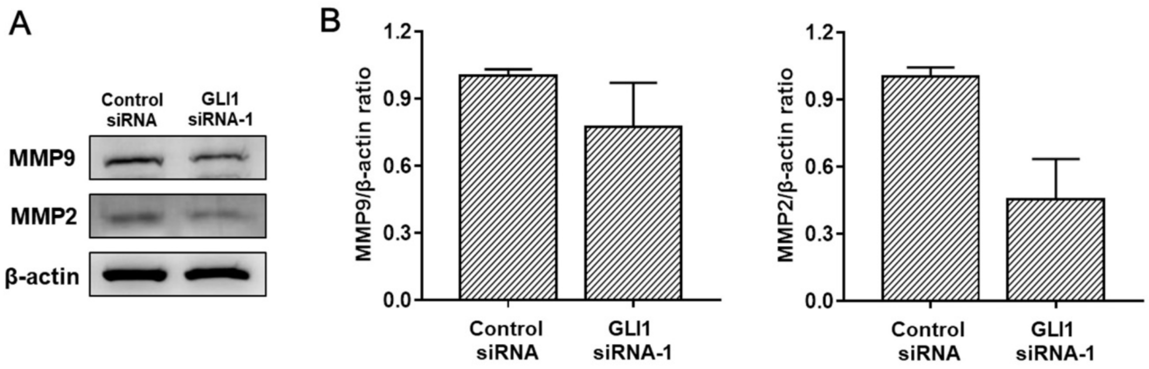

Supplementary Figure 2. Effect of GLI1 knockdown on the expression of MMP2 and MMP9 in MDA-MB-231 cells. (**A**) Immunoblots of MMP2 and MMP9 protein expression in MDA-MB-231 cells transfected with GLI1 siRNA or control siRNA for 48 h. GLI1 siRNA knockdown in MDA-MB-231 cells results in reduced expression of MMP2 and MMP9 levels. The blots were stripped and reprobed for *β*-actin as an internal control for equal loading. (**B**) Data indicate values performed in triplicate and the fold difference relative to *β*-actin. Data are shown as the means ± standard deviation (SD) of triplicate experiments.
